# The Role of Diisocyanate Structure to Modify Properties of Segmented Polyurethanes

**DOI:** 10.3390/ma16041633

**Published:** 2023-02-15

**Authors:** Manuel Asensio, Juan-Francisco Ferrer, Andrés Nohales, Mario Culebras, Clara M. Gómez

**Affiliations:** 1 Institute of Materials Science, University of Valencia, 46980 Paterna, Spain; 2 Plastics Technology Centre (AIMPLAS), 46980 Paterna, Spain; 3 R&D Department UBE CORPORATION EUROPE, S.A., 12100 El Grao, Spain

**Keywords:** thermoplastic polyurethane, diisocyanate, hard segment, phase segregation, mechanical and thermal properties

## Abstract

Segmented thermoplastic polyurethanes (PU) were synthetized using a polycarbonatediol macrodiol as a flexible or soft segment with a molar mass of 2000 g/mol, and different diisocyanate molecules and 1,4-butanediol as a rigid or hard segment. The diisocyanate molecules employed are 3,3′-Dimethyl-4,4′-biphenyl diisocyanate (TODI), 4,4′-diphenylmethane diisocyanate (MDI), 4,4′-Methylenebis(phenyl isocyanate) 1-isocyanato-4-[(4-phenylisocyanate)methyl]benzene and 1-isocyanate-4-[(2-phenylisocyanate) methyl]benzene (ratio 1:1) (MDIi), isophorone diisocyanate (IPDI), and hexamethylene diisocyanate (HDI). The polyurethanes obtained reveal a wide variation of microphase separation degree that is correlated with mechanical properties. Different techniques, such as DSC, DMA, and FTIR, have been used to determine flexible–rigid segment phase behavior. Mechanical properties, such as tensile properties, Shore D hardness, and “compression set”, have been determined. This work reveals that the structure of the hard segment is crucial to determine the degree of phase miscibility which affects the resulting mechanical properties, such as tensile properties, hardness, and “compression set”.

## 1. Introduction

Nowadays, polyurethanes (PUs) are one of the main synthetic polymer materials used worldwide due to their large range of applications, such as production of synthetic rubbers, adhesives, foams, fibers, protective coatings, elastomers, semi-permeable membranes, rigid devices, and sealants [1,2,3,4]. Their structure is characterized by the presence of a high proportion of urea and/or urethane linkages. The composition of the polyurethane polymer backbone is crucial to define the final properties of the material (mechanical performance, temperature resistance, optical properties, and processing conditions). Therefore, due to the large versatility in terms of molecular composition, polyurethanes can be specifically prepared for every application, from foams to rigid materials. In particular, segmented thermoplastic elastomer PUs are block copolymers consisting of alternating flexible or soft and hard or rigid segments, giving two phases a separated structure that is responsible for the final properties [4]. Typically, the flexible segment is a macrodiol with a low glass transition temperature, including polyethers, polyesters, and polycarbonates. Polyester polyol imparts high mechanical strength, polyetherdiol one gives high resistance to hydrolysis, and polycarbonate polyol enhances thermal stability of PU. Polyester and polyether polyols are the most used as flexible segments due to the low price and easy handling, while polycarbonate polyol is employed for specific and high value PU applications. The hard segment is formed by a diisocyanate and a chain extender. The chain extender is a low-molecular-weight difunctional intermediate designed to react with isocyanate groups. Aromatic and aliphatic diols and diamines can act as chain extenders. In the case of diols, the hard diisocyanates are characterized by the percentage of NOC content and their functionality [5]. Three different types of diisocyanates are commonly employed to obtain PUs: aliphatic, aromatic, and cycloaliphatic. Aliphatic and cycloaliphatic diisocyanates are less used than aromatic ones. The reasons are because the aromatically linked isocyanate group is much more reactive than the aliphatic one and because they are cheaper. Although a lot of work has been published on aromatic-derived PUs, a comparison of PUs built up with the different types of diisocyanates keeping the other components and concentrations constant is missed and would help to gain scientific knowledge of PU properties [6].

The flexible segment is responsible for the elasticity and flexibility of the PUs at room temperature, while the rigid segment provides mechanical strength to the PUs due to the large number of hydrogen-bonding interactions, which form a physically cross-linked network. The molecular interactions in both segments are crucial to understand the final properties of PUs. The elastomeric behavior of these materials is extremely related to the microphase separation of rigid–flexible segments. Thus, the final properties are based on the composition of the starting blocks used during the synthesis. Because the composition of the soft and hard phases can present a large variety of structures, PUs can exhibit a myriad of properties. Moreover, PUs can be obtained from different production procedures (with or without solvent, casting, injection, reactive extrusion, spraying) to make goods with different sizes and shapes. Another crucial factor in this type of PU is the hydrogen bonds between the hard and/or soft segments, which allows melt processing at certain temperatures when these interactions are broken. In addition, a great advantage is that they can be easily recycled [1,2,3,4,7,8,9].

In previous work, we have demonstrated how the composition of the flexible segment in thermoplastic elastomer polyurethanes allows the possibility of tuning properties depending on the desired application [10,11,12,13]. However, not only the flexible segment is crucial to define the final properties, but also the molecular structure/composition of the hard segment. This domain, when using a polyol as chain extender, contains the urethane groups derived from the reaction between isocyanate and hydroxyl groups. When rigid segments segregate from the flexible segment, the final PU increases its degree of crystallinity. However, the crystallinity also depends on other factors, such as the symmetry or the nature of both the diisocyanate and the chain extender, as well as the preparation procedure [4,12,14,15,16,17]. The melting point of the final PU is also affected by the composition of the rigid segment; in particular, the melting temperature increases with the content of the rigid segment. Therefore, all these structural and formulation aspects need to be understood in order to control and optimize polyurethane composition and processing parameters.

Thermoplastic polyurethanes have been studied for a long time since their discovery. Research papers that compare similar systems are called into question, since when one of the components varies, either in structure or composition, the final properties vary significantly, and the conclusions cannot be extended to a more general system [12,13]. In this sense, our research group has been investigating the influence of the different raw components needed to build up a thermoplastic polyurethane network, such as the chain extender, diisocyanate and soft segment (molar mass, structure, final composition), in the final properties to obtain a guide to design PUs with selected properties [8,10,11].

The present study focuses on the synthesis of segmented thermoplastic elastomer PUs, varying the structure of the rigid segment to establish a full understanding of the structures–properties relationships, which are crucial for the applicability of PUs. Therefore, PUs were synthetized utilizing a polycarbonate diol as the flexible phase, and 1,4-butanediol as the chain extender, which, by reacting with different diisocyanates, gives the hard phase. Five different diisocyanates were utilized: aromatic 3,3′-Dimethyl-4,4′-biphenyl diisocyanate (TODI), 4,4′-diphenylmethane diisocyanate (MDI), 1-isocyanate-4-[(4-phenylisocyanate)methyl]benzene and 1-isocyanate-4-[(2-phenylisocyanate)methyl]benzene (1:1) (MDIi), cyclic and non-aromatic isophorone diisocyanate (IPDI,) and linear hexamethylene diisocyanate (HDI). The different PUs were characterized by several techniques, including DSC, FTIR, DMA, tensile stress, shore hardness, and “compression set”. This work represents a full, comprehensive study of crucial structural and processing parameters that establish clear links between synthetic aspects, processing conditions, and final properties, which are key for polymer application understanding in general and, more precisely, for the PU industry.

## 2. Materials and Methods

### 2.1. Materials

Several segmented polyurethanes have been synthetized, changing the molar structure of the diisocyanate. The flexible segment or macrodiol of average molar mass 2000 g mol^−1^ is poly(hexamethylene) carbonate diol Eternacoll^®^ UH (UH200) supplied by UBE Chemical Europe (Castellón, Spain). The rigid segment consists of five different diisocyanates: 3,3′-Dimethyl-4,4′-biphenyl diisocyanate (TODI), 4,4′-diphenylmethane diisocyanate (MDI), 4,4′-diphenylmethane diisocyanate (MDI), 1-isocyanate-4- [(4-phenylisocyanate)methyl]benzene and 1-isocyanate-4-[(2-phenylisocyanate)methyl]benzene (ratio 1:1) (MDIi), isophorone diisocyanate (IPDI), and hexamethylene diisocyanate (HDI) reacting with 1,4-butanediol (purchased from Sigma Aldrich (Barcelona, Spain)). All materials were used as received and kept in a dry box to avoid humidity.

The main characteristics, such as abbreviation molecular structure, molecular weight, glass transition, and melting temperatures T_g_ and T_m_, respectively, of the raw materials used in this paper are summarized in Table 1.

### 2.2. Polyurethane Synthesis

The prepolymer synthesis method was used to obtain the segmented thermoplastic polyurethanes [10,12]. The process consists of two steps. First, the prepolymer was synthesized by mixing the macrodiol and an excess of diisocyanate in a reactor at a temperature of 70 °C for 1 h in an argon atmosphere to form a prepolymer of polyol endcapped with diisocyanate groups. Later on, the chain extender, butanodiol, at a molar ratio NCO/OH = 1.03, was added to the prepolymer in a SpeedMixerTM Dac 600.1 FVZ mixer (Landrum, SC, USA) at room temperature for 1 min at 2250 r.p.m. The PU samples were produced pouring the PU solution on aluminum molds at 90 °C, and pressed at 50 bars and 100 °C for 24 h using a water-cooled hydraulic Carver press model 4128CE S/N 4128-220 (Wabash, IN, USA). The cooling procedure was carried out under a constant water flow rate. PU sheets were produced for future characterization requirements.

The polyurethanes synthetized were named as PU-X, X being the diisocyanate employed. Different molar ratios (UH200: diisocyanate: BD) according to Table 2 were used for the polyurethane synthesis in order to reach a 32 wt% of hard segment. Samples were tested in the as-molded condition only. The experimental results are the mean value of at least three independent tests.

### 2.3. Characterization

A TA Instrument Q20 (New Castle, DE, USA) equipped with a refrigerated cooling system and nitrogen purge was used to perform Differential Scanning Calorimetry (DSC) experiments. Indium was used to calibrate the equipment according to the manufacturer’s recommended procedures. An aluminum pan with 4–6 mg of sample was employed for every test. The uncertainty associated with each temperature is approximately ±2 °C. The samples were scanned twice from −80 °C to 220 °C at a heating rate of 20 °C min^−1^. The midpoint of the heat capacity change has been chosen to represent T_g_, the endotherm peak temperature is the T_m_, and the area of the endotherm peak is the variation in enthalpy, ΔH, associated with the melting process.

To perform Dynamic Mechanical Analysis (DMA), a 2980 Dynamic Mechanic Analyzer (TA instruments) equipped with tensile head and reducing force option using the Custom Test and single cantilever geometry was employed. Calibration was performed according to the recommendations of the manufacturer The dimensions of the samples were t (18.000 × 6.000 × 2.000) mm^3^. The samples were tested from −100 °C to 180 °C at a frequency of 1 Hz and amplitude of 30 µm (linear viscoelastic region) with a temperature ramp of 3 °C min^−1^. These experiments yield the storage modulus (E′), the loss modulus (E″), and the damping factor tan δ (=E″/E′). The glass transition temperature was determined from the peak of the tan δ curve.

A Thermo Nicolet Nexus FTIR spectrometer (Waltham, MA, USA) equipped with a multiple internal reflection accessory ATR single bounce was used to perform Fourier Transform Infrared-Attenuated Total Reflection Spectroscopy (FTIR-ATR) measurements. Samples were pressed against the ATR accessory diamond crystal by means of a fixing screw using a flat tip. Single beam spectra of the samples were obtained after averaging 128 scans between 4000 cm^−1^ and 400 cm^−1^ with a resolution of 4 cm^−1^. All spectra were obtained in the transmittance mode.

Shore A hardness was measured at room temperature using a Zwick Roell (Ulm, Germany) analogical hardness testing apparatus following the “UNE-EN ISO 868:1998: Plastics and ebonite. Determination of indentation hardness by means of a durometer (Shore hardness)” standard procedure at (23 + 2) °C and 50% relative humidity.

Tensile properties were measured at 23 °C on five replicates of each material with an Instron Model 5582 Universal Testing machine (Grove City, PA, USA) according to “ISO 527-3 Testing method for thermoplastic polyurethane elastomers”. A 10 kN load cell was used and the cross-head speed was 200 mm min^−1^. Pneumatic grips were required to hold the test specimens.

The “compression set” measurements were carried out following the ISO 815 standard under different conditions to determine the retention of elastic properties after prolonging a constant compression stress at certain temperatures. The samples for this test are cylindrical, with a diameter of (2.90 ± 0.05) mm and (12.5 ± 0.5) mm height. The tests are carried out after 72 h of compression at 70 °C and 100 °C. The value of “compression set” as a percentage is obtained from the following equation:

(1) $$\mathrm{Compression}\;\mathrm{set}\;\%=\frac{h_{i}-h_{f}}{h_{i}-h_{c}}\cdot 100$$

where h_i_ is the initial height of the sample, h_f_ is the height of the sample at the end of the test, and h_c_ is the constant height to which the sample is subjected during the test.

## 3. Results and Discussion

This paper focuses on the properties’ influences on the molecular structure of the diisocyanate that forms part of the rigid segment of thermoplastic segmented polyurethanes. Five different polyurethanes have been synthetized without solvent by the two-step method, with polycarbonate diol UH200 as the flexible segment and butanediol and five different diisocyanates as the rigid segment.

The influence of the rigid segment molecular structure on the morphology and thermal and mechanical properties has been investigated. The main objective of this study is to evaluate the influence of the diisocyanate in the hard phase in order to tailor the properties of polyurethanes based on a specific application. The utilized diisocyanates change the molecular structure from linear (HDI) to cyclic (IPDI) to aromatic (TODI, MDI, MDIi).

**Figure 1:**
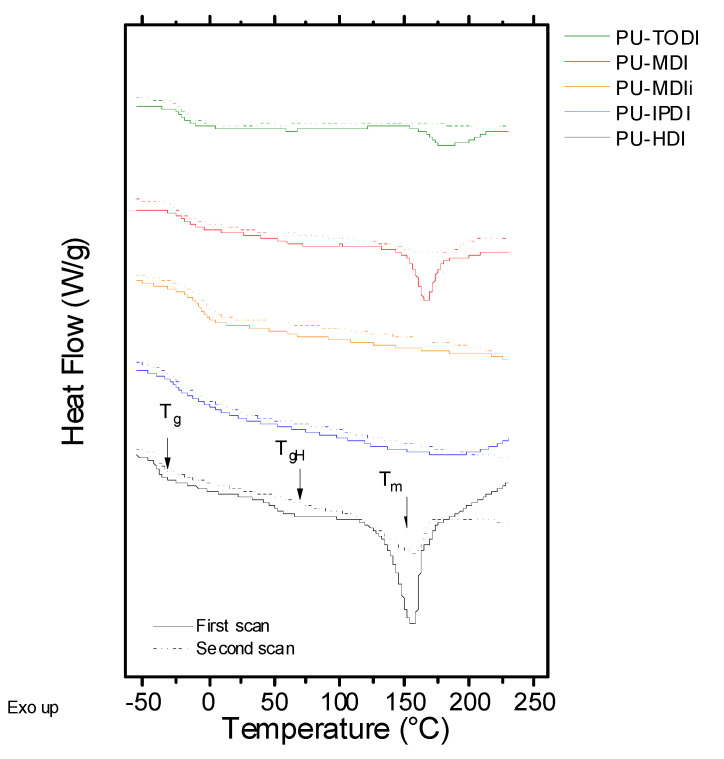
DSC curves for the different polyurethanes studied showing the characteristic transitions during the first (continuous line) and second scans (dashed line).

Differential scanning calorimetry (DSC) curves have been obtained to determine the behavior under heat flow. Figure 1 depicts the first and the second scans of all the samples assayed as a function of temperature. All the systems show two temperature regions: the one at lower values shows glass transition and the one at higher temperatures shows an endothermic peak related to the melting process of the crystalline domains of the PU. The glass transition temperature observed at low temperature values is related to the amorphous part of the flexible segment. Values of T_g_ of the polyurethane strongly depend on the type of macrodiol utilized in the synthesis, in this case UH200, and the interactions with the rigid segment. In the case of the PUs developed, the T_g_ is in the range of (−40)–(−15) °C, indicating that the structure of the rigid segment plays an important role during the phase separation between the flexible and rigid segments. Moreover, for the samples based on MDIi and HDI, a transition can be clearly observed around 60 °C, which is related to the glass transition temperature of the rigid segments, T_gH_. This transition will depend on two factors: the glass transition temperature of the pure rigid segments and the degree of phase segregation between flexible and rigid segments [18,19]. Finally, for materials that can establish a high ordering degree of the rigid segment (crystallinity), an endothermic peak, T_m_, is observed, which is located around 150 °C [20,21,22,23]. This fact is clearly evidenced in PUs consisting of HDI, TODI, and MDI.

In order to determine the degree of mixing macrodiol or the flexible segment with the rigid segment, values of the difference between the glass transition temperature of the polyurethane (T_g_ in Table 3) and the T_g_ of the macrodiol (T_g,S_ in Table 1), (T_g_−T_g,S_) are compiled in Table 3. Based on the glass transition temperatures of the polyurethanes shown in Table 3, it is possible to give an idea about the interaction degree between the PU chains in both types of segments. The miscibility between the flexible phase and the rigid phase is determined by the difference between the glass transition temperature of the polyurethane, T_g_, and the glass transition temperature of the pure macrodiol utilized, T_g,S_ [10,24,25,26]. The smaller the difference is between these two temperatures, Δ(T_g_−T_g,S_), the greater will be the phase segregation between both segments. Thus, the miscibility order, from lower to higher, is HDI < TODI < MDI < IPDI < MDIi. This trend is explained by the ability to form crystalline domains with the rigid segments. Therefore, for the highly crystalline segment based on HDI, TODI and MDI, the miscibility is lower.

Additionally, the T_g_ can be related to the fraction of the hard segment in the soft phase w_H,DSC_ due to the fact that the change in thermal properties of a two-component system (hard and soft segments) is the linear addition of the change in the property of the two components. This dependence can be expressed with the equation obtained by T. K. Chen et al. [27]

(2) $$T_{g}=\left(1-w_{H,\mathrm{DSC}}\right)T_{g,S}+\left(w_{H,\mathrm{DSC}}\right)T_{g,H}$$

where T_g,H_ is the glass transition temperature of the rigid segment. These glass transition temperatures were obtained from synthesized polyurethanes formed only by a hard segment with a molar ratio of 1:1 diisocyanate: BD. The T_g,H_ obtained can be related to the degree of interaction experienced by the hard segments in the different polyurethanes. The increment of this temperature is a consequence of a low degree of interaction with fragments of the flexible segment due to fact that the interaction between rigid segments is more favorable in creating crystalline domains.

As seen in Table 3, values of w_H,DSC_ correlate with the order of miscibility obtained by the T_g_ increment, and higher miscibility results in higher w_H,DSC_ and a higher percentage of hard segment in the soft phase. For the case of PUs synthesized with aromatic diisocyanates (MDI and TODI), the values of w_H_ are higher than the ones obtained for PU based on HDI. This fact is related to the structure of rigid segments given by the diisocyanate utilized having a lower ordering degree (low ability to form crystals) due to the structure of the molecule. This is clearly evidenced in the case of PUs obtained from TODI, where the -CH_3_ substituents in each of the rings’ aromatics hinders the interaction between urethane groups through hydrogen bonding [11,24]. In addition, in the case of highly isometric diisocyanates, the values of w_H_ are the highest, indicating their poor ability to form crystalline domains and, consequently, increasing the miscibility of the hard segments in the flexible segments.

**Figure 2:**
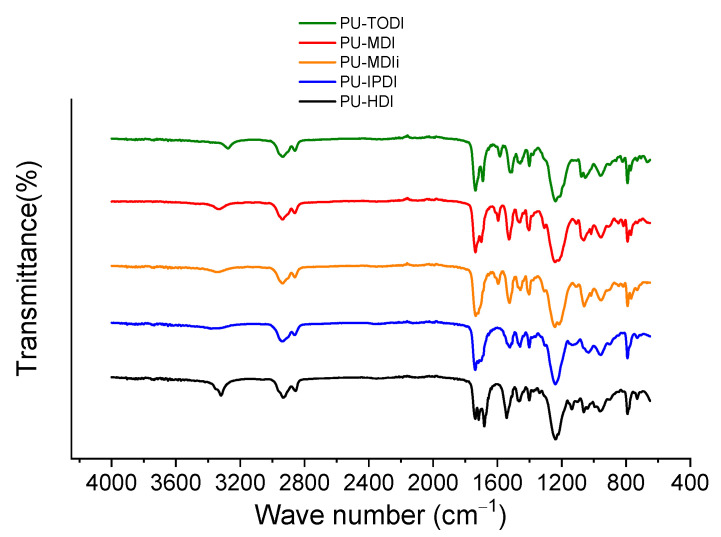
Fourier transform infrared-attenuated total reflection spectra showing transmittance spectra of the different polyurethanes.

The structural aspects of PUs were evaluated utilizing infrared spectroscopy. Figure 2 shows the FTIR spectra of the PUs prepared. The main bands commonly used to characterize segmented PUs are the OH absorption at 3500 cm^−1^, the NH stretching vibration at 3500–3000 cm^−1^, and CO group of urethane at 1800–1640 cm^−1^. The band at 3276 cm^−1^ evidences the formation of NH of the urethane linkage, while the –OH band at 3470 cm^−1^ of the corresponding polyols disappears. Simultaneously, the NCO stretching band at 2273 cm^−1^ disappeared as a consequence of the reaction between OH and NCO groups. These facts reveal the formation of PU consistent with the reaction of –OH with –NCO [13].

The carbonyl absorption region (1630–1730 cm^−1^) is particularly interesting because it gives information about inter-urethane hydrogen bonding. The assignation of each band in the carbonyl regions is summarized in Table 4.

**Figure 3:**
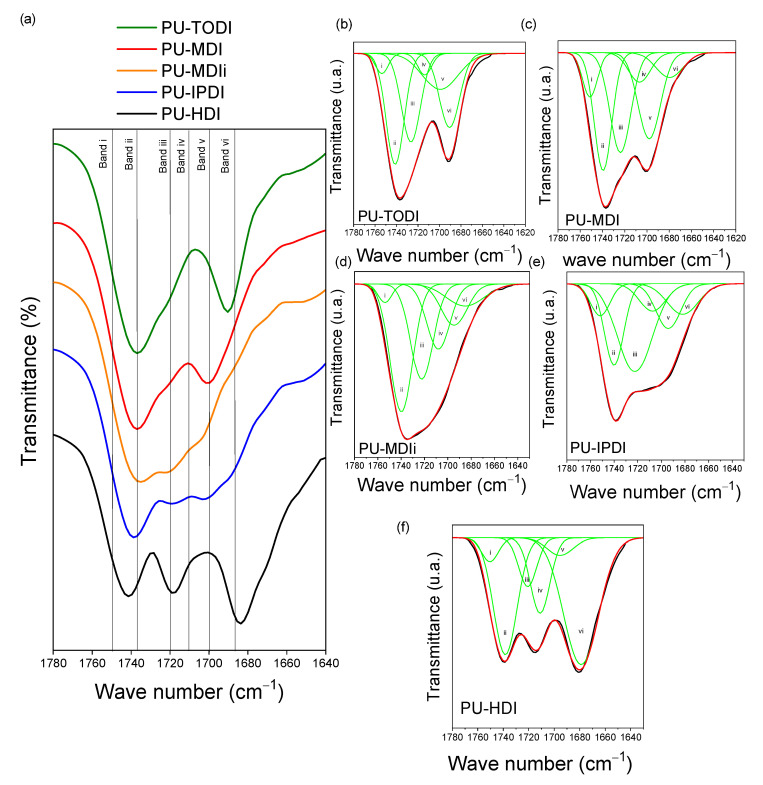
(**a**) FTIR bands in the region of the carbonyl group for polyurethanes based on the different diisocyanates. (**b**–**f**) deconvoluted spectra for the different PUs produced.

Figure 3 shows the FTIR bands corresponding to the carbonyl group region for every PU. These bands show significant differences in terms of the diisocyanate used during the synthesis. A more detail study is provided by deconvolution of the carbonyl band (Figure 3b–f). For all the materials, band ii is related to the associated carbonyl groups of the flexible segment, which appears in a predominant manner (1740 cm^−1^), while the other bands are present in different ratios, evidencing important structural differences between the PUs prepared.

The contribution (in %) to the total carbonyl absorption of band i, band ii, band iii, band iv, band v, and band vi is shown in Table 5.

According to the values shown in Table 5, band ii, which is related to the carbonyl groups of the flexible segment associated with each other, presents a greater area than band i (related to the free flexible segment carbonyl groups), indicating a certain degree of association between carbonyls belonging to the flexible segment. Band iii is related to the non-associated carbonyl groups in the rigid segments. For the case of PUs based on MDIi and IPDI, these values are higher than for the rest of the PUs, evidencing that the interaction between rigid segments is more impeded in these materials. Band iv (located at 1711 cm^−1^) represents the associated carbonyl groups between the rigid and flexible segments. For the case of the HDI-based PU, the contribution of this band to the total absorption of the carbonyl region is the highest, indicating that some of the rigid segments tend to interact with the flexible segments. Bands v and vi are related to the way in which the polymeric chains organize the rigid segments. Except for HDI, all materials present an important contribution related to the formation of disordered rigid segment phases (v band).

To quantify the degree of miscibility between phases, the weight fraction of the rigid segment in the flexible segment, w_H,FTIR_, is used. This parameter is obtained from the following equation, formulated by Sung and Schneider [26]:

(3) $$w_{H,\mathrm{FTIR}}=\frac{{\left(1-X_{b}\right)}_{\zeta }}{\left[{\left(1-X_{b}\right)}_{\zeta }+\left(1-\zeta \right)\right]}$$

where the factor ζ indicates the total ratio of the rigid segment in the materials. In the present study, it was 0.32 for all the samples. X_b_ indicates the fraction of carbonyl groups of urethanes linked by hydrogen bonds, which can be obtained by the following equation:

(4) $$x_{b}=\frac{\mathrm{Band}\;\mathrm{vi}\left(\%\right)+\mathrm{Band}\;v\left(\%\right)}{k\left(\mathrm{Band}\;\mathrm{iii}\left(\%\right)\right)+\mathrm{Band}\;\mathrm{vi}\left(\%\right)+\mathrm{Band}\;v\left(\%\right)}$$

where k is a constant representing the ratio of the absorption coefficient of bound and free urethane carbonyl groups. In this work, a value of 1.2 has been used [18,28]. Table 6 shows the values of X_b_ and w_H_,_FTIR_, calculated using Equations (3) and (4).

The values shown in Table 6 present different degrees of miscibility between phases for the samples produced. High values of X_b_, which is the fraction of associated urethane groups, and low values of w_H,FTIR_, the weight fraction of the rigid phase contained in the flexible phase, indicate a higher value of segregation between phases [27]. According to the values obtained, the highest degree of miscibility between phases is obtained for materials based on diisocyanates such as MDIi and IPDI, with W_H,FTIR_ values of 0.23 and 0.22, where around 70% of the rigid segment interacts with the flexible segment. In these materials, based on MDIi and IPDI, the rigid segments have greater structural impediments to easily associate with each other. Both TODI-based and MDI-based materials present intermediate miscibility values of 0.19 and 0.20, respectively, with percentages of the rigid segment in the flexible segment of around 6%, similar to the data obtained in previous reports [28]. In addition, we found lower miscibility values for HDI-based materials, where the association between rigid segments is favored due to their type of structure when compared to the other isocyanates used in the present study [29].

**Figure 4:**
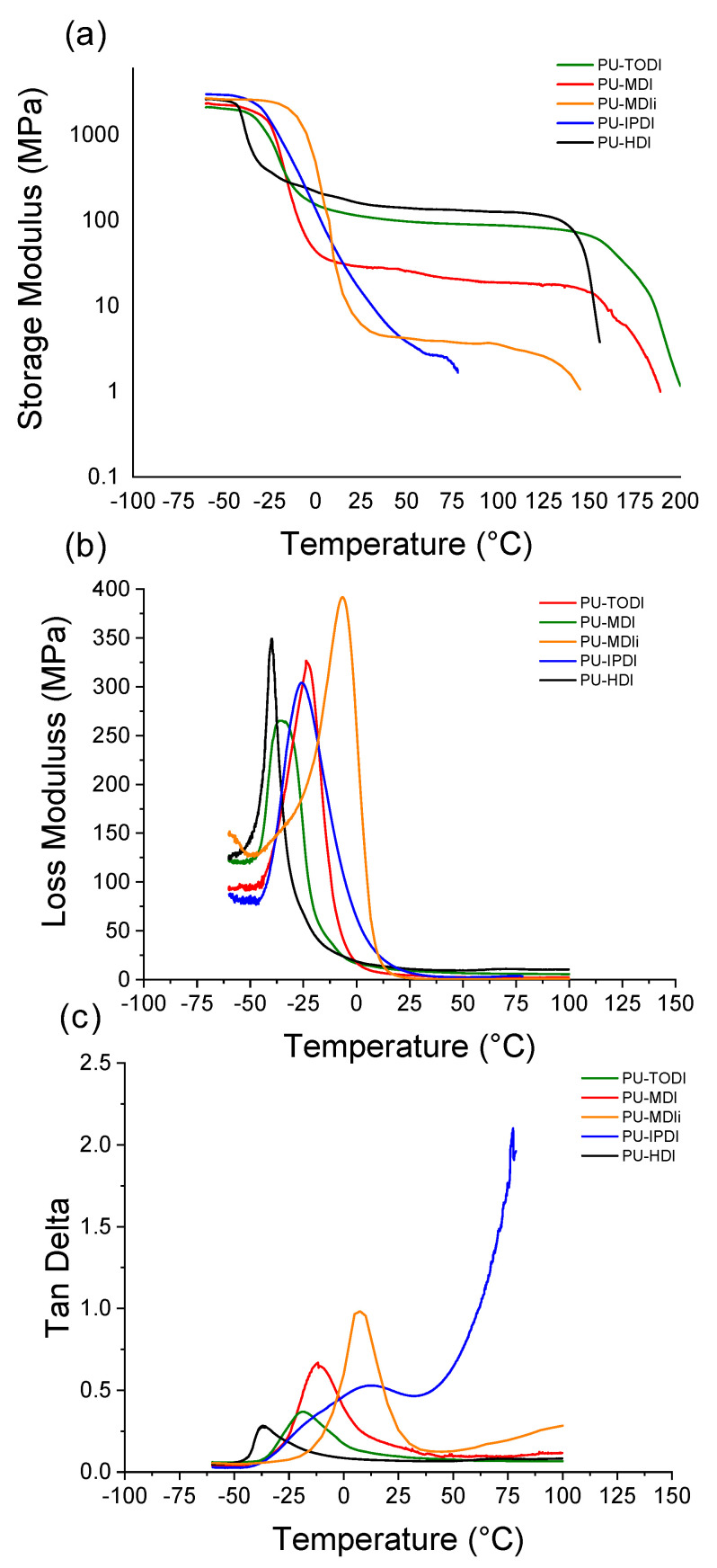
Data obtained from DMA measurements: (**a**) Storage modulus (E′), (**b**) loss modulus (E″) and (**c**) tan δ of materials based on the different diisocyanates.

DMA has been used to obtain information on the viscoelastic properties that can be related with soft and hard microdomain thermal transitions [20,30,31]. Figure 4 shows the curves obtained for the storage modulus as a function of temperature for all the synthesized polyurethanes. For all PUs, the curves of the storage modulus (E′) show a vitreous zone at low temperature, since the materials are in a vitreous state and are capable of absorbing more mechanical energy than at higher temperatures [24,30,31,32,33]. As the temperature increases, the storage modulus decreases, reaching the glass transition temperature, and the mechanical energy storage capacity of the material decreases until reaching the elastomeric region, where E’ remains practically constant. Finally, at higher temperatures, there is a sudden drop in storage modulus, indicating the material softens and the modulus decreases.

For the case of the loss modulus curves, E″, there is a significant increase for the temperature region in which the transition between the glassy and the viscoelastic zone occurs. In this zone, the PUs have reached the glass transition temperature and are capable of dissipating energy due to the fact that the chains present some sort of degree of mobility and are capable of interacting with each other through the mechanical stress produced by the test. As the temperature increases, the loss modulus decreases because the material reaches the viscoelastic zone and requires less energy to maintain a constant displacement. The tan δ curve is obtained from the ratio E″/E′, so it measures the ratio of energy dissipated/energy absorbed. In this curve, a peak is observed, related to the T_g_ of the materials, that subsequently decreases at that temperature due to the decrease observed in the loss modulus [34,35].

In the case of the storage modulus, as the miscibility between phases is lower, the corresponding decrease in the storage modulus related to the T_g_ will occur at a lower temperature interval, due to the fact that there is less rigid segment inclusion in the flexible segment inhibiting the movement of the chains [34]. In the case of the materials studied, the miscibility values have been calculated by DSC and FTIR, obtaining values of the rigid segment in the flexible segment depending on the isocyanate used, which follow the trend PU-HDI < PU-TODI < PU-MDI < PU-MDIi < PU-IPDI.

Regarding the decrease in the storage modulus of each material, it can be seen, in Figure 4a, how the temperature interval in which it occurs follows the same trend in terms of the miscibility values between phases observed by DSC and FTIR. Thus, HDI-based materials, which experience greater phase segregation, undergo a transition between the glassy region and the elastomeric region that is smaller compared to materials with higher miscibility values, such as the case of IPDI-based materials.

The average width of the peaks obtained in the loss modulus curves that appear in Figure 4b are related to the temperature interval for the drop of the storage modulus, making it possible to quantify the temperature range for the transition from the vitreous zone to the elastomeric zone [34]. Figure 4c shows the curves obtained for Tan δ.

Table 7 shows the storage modulus values as a function of temperature, as well as the peak width values of the loss modulus, and the tan δ values obtained by DMA for the different materials.

Observing the values of the storage modulus as a function of temperature, a non-linear, and different, decrease can be seen in the different materials. In the case of materials with greater phase segregation, observed by DSC and FTIR (the ones based on HDI and, to a lesser extent, based on TODI and MDI), a drop in the storage modulus around 12% was observed between 25 and 50 °C. For materials based on MDIi and IPDI, E′ decreases around 28% and 74%, respectively, for the mentioned temperature range. This means that the greater phase segregation causes the chains to be better able to store energy at temperatures higher than room temperature, while in materials with less phase segregation, where the chains are less organized, they are not capable of storing energy in the same way [24,25].

The width of the peaks in the loss moduli of the materials (see Table 7) reflects a transition between the vitreous zone and the elastomeric zone. This transition is greater for materials with lower phase segregation, such as those based on MDIi or IPDI, with values of 76.85 °C and 74.68 °C, respectively. The lowest peaks are obtained for materials based on HDI, TODI, or MDI, which indicates a lower amount of rigid segment in the flexible segment, which causes greater mobility in the regions of the flexible segment that govern the change between the vitreous zone and the elastomeric zone [21].

Regarding the values obtained in the tan δ curves, peaks are observed whose maximum is related to the Tg of the materials, following the trend observed in the DSC curves [36], and in which higher temperatures can be seen for materials that suffer less phase segregation.

**Figure 5:**
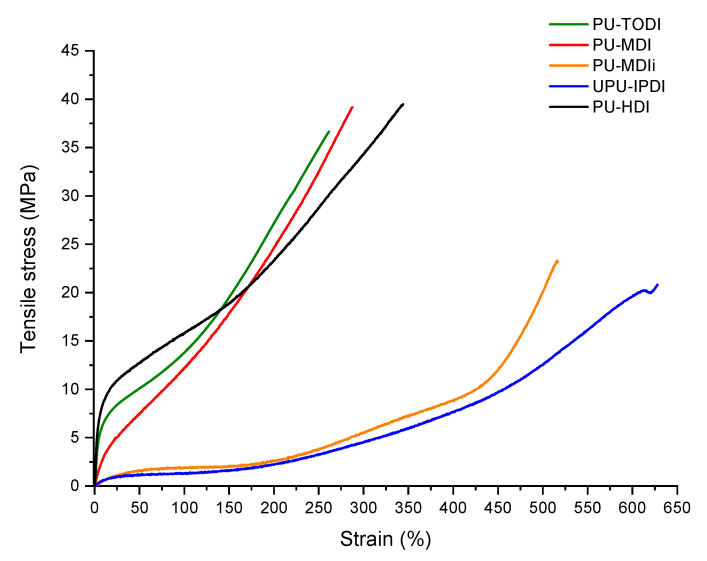
Tensile stress curves for the different PU samples at 25 °C.

The tensile stress/strain curves are shown in Figure 5. The PUs based on TODI, MDI, and HDI present higher values of tensile stress than the samples produced with IPDI and HDI. In contrast, higher strain values were obtained for those PUs. As was shown before in the DSC and FTIR results, HDI-based materials presented higher segregation values, and the rigid segments formed with the chain extender are translated into more linear and ordered polymer chains, which is the reason for greater tensile stress at low deformation values.

The samples prepared with TODI and MDIi present lower phase segregation values, so there is a greater inclusion of the rigid segment into the flexible segment, which makes it difficult for the rigid segment domains to handle the tensile stress compared to the HDI-based materials. Between these two materials, despite having similar miscibility values, there are differences in terms of elastic modulus values. In this case, the TODI-based materials have more linear rigid segments compared to the MDI-based ones, so they depicted higher tensile strength at low deformation values.

For PUs based on IPDI and MDIi, although the materials exhibit lower tensile strength values, their ability to deform is greater compared to the samples prepared with TODI, MDI, or HDI. Table 8 shows the average values of tensile stress at 100%, 200%, and 300% deformation, tensile stress at break, and the elongation at break obtained in the tensile stress analysis.

The results show clear differences depending on the isocyanate used. For the PUs with a better phase miscibility (such as those based on MDIi or IPDI), the polymeric chains are more disordered, so the rigid segments that govern the tensile strength are not able to handle the mechanical stress of the PUs well with ordered rigid domains [37]. In contrast, they offer high strain values, since these materials present more urethane–urethane hydrogen bond interactions, which are less intense.

Higher tensile stress values are obtained for PUs with higher phase segregation (HDI, TODI, and MDI) since the hard segment phase is better spatially ordered, and thus exerts greater tensile strength. On the contrary, these more ordered structures are not capable of withstanding high elongation values. From 200 elongation, the more disordered nature of the flexible segments is the one that dominates the tensile strength. PUs based on MDIi and IPDI, with higher miscibility between soft and hard segments, cause significantly higher elongation values.

Table 9 shows the hardness and compression set values obtained for the different PUs produced. In the case of the hardness, the values obtained are higher for the PUs based on HDI, TODI, and MDI compared to PUs based on MDIi or IPDI. This fact is explained because HDI, TODI, and MDI lead to a final PU with a higher elastic modulus compared to MDIi and IPDI. These hardness values reveal a greater molecular interaction within the rigid segment that derives from a greater crystallinity, which makes these materials more resistant to penetration [38].

The behavior against a constant compression in this type of material depends mainly on the nature of the rigid segment [39]. Therefore, lower compression set values are expected for the one based on HDI, followed by those based on TODI, MDI, MDIi, and IPDI. However, the lowest values correspond to materials with MDI and TODI. This fact is evidence that, in the case of PUs based on HDI, the lower miscibility between phases allows a high content of the flexible segment which does not interact with the hard segment, thus decreasing the number of interactions between polymer chains compared to materials based on TODI and MDI, where the phase segregation is lower, allowing a greater recovery of the initial shape. Both the MDIi- and IPDI-based materials did not return to shape in either of the two tests. The greater miscibility of phases in these materials causes a very low stiffness that does not allow the materials to resist constant compression.

## 4. Conclusions

Several PUs with different rigid segments derived from diisocyanates based on TODI, MDI, MDIi, IPDI, and HDI have been produced. The DSC and FTIR analysis showed a miscibility trend of PU-HDI < PU-TODI < PU-MDI < PU-MDIi < PU-IPDI, indicating that the type of molecular structure of the diisocyanate is crucial for the interactions between segments and phase separation. The storage modulus and the maximum of tan δ depict higher values in the same order than the increase in miscibility, indicating higher stiffness and phase segregation. This behaviour is tightly related with the higher degree of hydrogen bonding in the rigid segments, responsible for a greater stability in the viscoelastic region in these materials. The mechanical properties are aligned with this trend due to the fact that materials with greater phase miscibility and a lower intermolecular bonding degree lead to higher elongation values, since they have a more amorphous morphology and disordered chain orientation. In contrast, the amorphous nature causes a lower interaction degree between polymer chains, which is translated into lower tensile values. The present study reveals the good relationship between hydrogen bonds, phase miscibility, phase segregation, and mechanical properties in PU systems. So it can be concluded that low miscibility and high phase segregation of hard and soft segments bring, as a consequence, high tensile stress, low elongation at break, and good hardness and compression set values.

## Figures and Tables

**Table 1:** Abbreviation, structure, molecular weight, and T_g_ and T_m_ of the raw materials used.

Abbreviation	Structure	Molecular Weight (g mol^−1^)	T_g_ (°C)	T_m_ (°C)
UH200	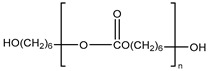	2000	−52.30	50.4
BD	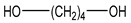	90.12	-	20.1
TODI	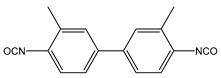	264.28	-	72
MDI	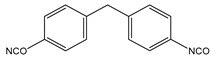	250.26	-	40
MDIi	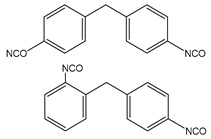	250.26	-	14
IPDI	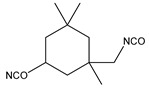	222.24	-	−60
HDI	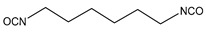	168	-	−67

**Table 2:** Symbol and molar ratio UH200:diisocyanate:BD of the different PUs developed.

Symbol	Molar Ratio
PU-TODI	1:2.9:1.9
PU-MDI	1:3:2
PU-MDIi	1:3:2
PU-IPDI	1:3.3:2.3
PU-HDI	1:4:3

**Table 3:** Values of the glass transition temperature (T_g_), temperature difference (T_g_−T_g,S_), glass transition temperature of the rigid segment, T_g,H_, fraction of rigid segment in flexible segment, w_H,DSC_, and percentage of rigid segment in flexible segment of all polyurethanes.

	T_g_ (°C)	Δ(T_g_−T_gS_) (°C)	T_g,H_ (°C)	w_H,DSC_	Percentage of Hard Segment in Soft Segment (%)
PU-TODI	−21.8	28.25	108.2	0.18	5.7
PU-MDI	−20.2	29.85	111.4	0.18	5.9
PU-MDIi	−16.9	33.15	99.5	0.22	7.1
PU-IPDI	−17.9	32.15	98.6	0.22	6.9
PU-HDI	−38.7	11.35	64.1	0.10	3.2

**Table 4:** Assignation of the FTIR wave numbers to the different contributions of the carbonyl group band.

Contribution to Carbonyl Band	Wave Number (cm^−1^)
Free carbonyl groups (flexible segment)	1750 (band i)
Associated carbonyl groups (flexible segment)	1736 (band ii)
Carbonyl groups of unassociated urethane groups (rigid segment)	1720 (band iii)
Associated carbonyl groups (Seg. Rigid-Seg. Flexible)	1711 (band iv)
Associated carbonyl groups (Sec. Rigid-disordered structure)	1699 (band v)
Associated carbonyl groups (Sec. Rigid-ordered structure)	1688 (band vi)

**Table 5:** Contributions of the deconvoluted signals for the carbonyl region for each material.

	PU-TODI	PU-MDI	PU-MDIi	PU-IPDI	PU-HDI
Band i (%)	6.11	6.74	5.22	4.45	5.85
Band ii (%)	36.04	37.62	36.62	34.18	28.56
Band iii (%)	16.92	17.82	21.42	21.18	11.93
Band iv (%)	10.16	9.36	16.05	14.72	18.43
Band v (%)	26.78	20.91	14.84	11.78	4.29
Band vi (%)	3.99	7.55	5.85	13.70	30.93

**Table 6:** Fraction of associated urethane carbonyl groups, X_b_, weight fraction of rigid segment in the flexible phase, w_H,FTIR_, and percentage of rigid segment included in the flexible segment for all materials.

	PU-TODI	PU-MDI	PU-MDIi	PU-IPDI	PU-HDI
X_b_	0.51	0.48	0.36	0.42	0.71
W_H,FTIR_	0.19	0.20	0.23	0.22	0.12
% of rigid Segment	6.1	6.4	7.4	7.0	3.8

**Table 7:** Storage modulus values as a function of temperature and maximum temperature values of tan δ and loss modulus peak width for each PU.

	Storage Modulus (MPa)			Loss Modulus (MPa)	Tan δ	
	25 °C	50 °C	100 °C	Peak Width (°C)	Maximun	Maximun Temperature (°C)
PU-TODI	112.1	97.3	87.7	69.48	0.37	−18.7
PU-MDI	28.9	25.7	18.9	67.3	0.67	−11.8
PU-MDIi	5.9	4.2	3.8	76.85	1.10	6.03
PU-IPDI	15.1	3.8	-	74.68	0.53	12.9
PU-HDI	158.5	140.0	126.2	46.68	0.28	−36.5

**Table 8:** Mechanical properties of the segmented polyurethanes at 25 °C.

System	Tensile Stress at 100% (MPa)	Tensile Stress at 200% (MPa)	Tensile Stress at 300% (MPa)	Tensile Strength at Break (MPa)	Elongation at Break (%)
PU-TODI	13.6 ± 0.4	26.9 ± 0.9	-	33 ± 3	240 ± 7
PU-MDI	11.16 ± 0.08	23.5 ± 0.2	-	31.6 ± 0.9	248+5
PU-MDIi	1.82 ± 0.06	2.5 ± 0.1	5.4 ± 0.2	24 ± 2	520 ± 26
PU-IPDI	1.6 ± 0.3	2.8 ± 0.5	5.4 ± 0.7	23 ± 2	620 ± 11
PU-HDI	15.9 ± 0.3	23.7 ± 0.4	35 ± 1	40 ± 5	360 ± 30

**Table 9:** Hardness and compression set values of the segmented polyurethanes.

	Hardness		Compression Set	
	Shore A	Shore D	70 °C 22 h	100 °C 72 h
PU-TODI	95	44	30	52
PU-MDI	94	42	20	50
PU-MDIi	69	29	100	100
PU-IPDI	72	30	100	100
PU-HDI	97	48	44	71

## Data Availability

No additional data.
